# Dynamic coronary roadmap-guided PCI reduces contrast volume and radiation time compared to standard angiography PCI: A meta-analysis

**DOI:** 10.1016/j.heliyon.2024.e41557

**Published:** 2024-12-30

**Authors:** Mohammad Al Hayek, Ibrahem A. Beshr, Mohammed S. Beshr

**Affiliations:** a Faculty of Medicine, Damascus University, Damascus, Syrian Arab Republic; b Faculty of Medicine and Health Sciences, Sana'a University, Sana'a, Yemen

**Keywords:** Percutaneous coronary intervention, PCI, Dynamic coronary roadmap, DCR-Guided, Angiography

## Abstract

**Background:**

Dynamic Coronary Roadmap (DCR) is a new PCI method that may reduce contrast dose and contrast-associated acute kidney injury (CA-AKI) risk. This paper evaluates DCR-guided PCI versus standard angiography PCI for contrast usage, procedure time, and CA-AKI risk.

**Methods:**

On May 1, 2024, we searched PubMed, Scopus, Embase, Cochrane Library, and clinicaltrials.gov for clinical trials or observational studies comparing DCR-guided PCI to standard angiography PCI. Outcomes were contrast media usage, radiation time, dose-area product, air kerma, radiation dose, post-PCI eGFR, AKI incidence, and procedure success. We used a random-effects model and analyzed outcomes using standardized mean difference (SMD) and odds ratio (OR).

**Results:**

Out of 1679 screened articles, only 5 were eligible, encompassing 941 patients. Findings show DCR-guided PCI significantly reduces contrast volume (SMD = −1.12, 95 % CI: 1.75 to −0.50, p = 0.0004), dose-area product (SMD = −0.71, 95 % CI: 1.25 to −0.17, p = 0.01), air kerma (SMD = −1.62, 95 % CI: 2.70 to −0.54), and radiation time (SMD = −0.75, 95 % CI: 1.32 to −0.18, p = 0.003) compared to standard angiography PCI. Despite lower incidence of acute kidney injury (AKI) in the DCR-guided PCI group, the odds ratio did not show statistical significance. Post-PCI eGFR also did not differ significantly between the two groups. Procedural success rates were similar, both exceeding 99 %.

**Conclusions:**

In this paper, we found that DCR-guided PCI is superior to conventional PCI in terms of contrast medium volume and radiation time. Future randomized controlled trials with larger sample sizes are needed to confirm these findings, especially in patients with kidney disease.

## 1 Introduction

Percutaneous coronary intervention (PCI) is a highly effective and successful treatment for coronary artery disease and it's been used widely in clinical practice. One of its downsides is the incidence of contrast-associated acute kidney injury (CA-AKI) among patients undergoing PCI, which is 13.3 % (95 % CI: 10.4–17.1). While the general population has a low risk of CA-AKI, ranging from 0.6 % to 2.3 %, high-risk subgroups may experience CA-AKI in up to 20 % of cases [1].

Risk factors such as older age, hypertension, diabetes mellitus, and a history of prior myocardial infarction increase the likelihood of its occurrence [2]. Moreover, in patients with chronic kidney disease (CKD), it escalates the risk of experiencing adverse events [3]. CA-AKI is associated with adverse outcomes such as prolonged hospitalization, irreversible kidney damage, dialysis dependency, and mortality [4].

Given the incidence of CA-AKI and its complications, the primary objective in clinical practice is to prevent its occurrence. One way to decrease the risk is by reducing the contrast volume during PCI, as it is the primary contributing factor to this problem [5,6].

A new approach with the potential to reduce contrast media dose is through performing PCI via Dynamic Coronary Roadmap (DCR) software. The DCR software, developed by Philips Medical Systems (Best, Netherlands), provides a live dynamic view of the coronary tree during fluoroscopy. It operates with a minimum image frequency of 7.5 pictures per second, generating a roadmap from a single angiogram, aiding PCI navigation. The software generates a coronary tree image to be used as a reference. It allows the operator to advance the wire using the map without the need for additional contrast to guide the wire. Eventually, it allows the navigation of PCI using a single contrast injection. Operators can store and access previous roadmaps, enhancing navigation and efficiency. This technology connects main vessels and side branches in ultra-low-dose contrast PCI, thereby reducing contrast usage and improving outcomes [7].

The aim of our study is to investigate whether using the DCR-guided PCI leads to a significant reduction in contrast volume compared to standard angiography PCI. We evaluated contrast media volume, dose-area product, air kerma, procedure success, post-PCI estimated glomerular filtration rate (eGFR) and incidence of acute kidney injury (AKI).

## 2 Materials and methods

We conducted this systematic review and meta-analysis following the Preferred Reporting Items for Systematic Reviews and Meta-Analyses (PRISMA) guidelines [8]. The PRISMA checklist is available in the supplementary files. The study protocol was registered in advance with PROSPERO under the registration number: CRD42024547001.

### 2.1 Literature search

On 1st of May 2024, we searched the following databases: PubMed, Scopus, Embase, Cochrane Library, and clinicaltrials.gov. We also conducted manual reference searching of key articles identified in PubMed. We used derivative keywords for the terms 'coronary dynamic roadmap,' 'percutaneous coronary intervention,' and 'contrast media' in our database search. A detailed overview of our search methodology, including the formulation of our search terms is presented in the supplementary file.

### 2.2 Study selection & data extraction

We used the Covidence platform (covidence.org, Melbourne, Australia) for screening articles. Titles/abstracts and full-texts screening were independently reviewed by two reviewers against our predefined inclusion and exclusion criteria. Any disagreements were resolved by the first author.

Our predetermined inclusion criteria were as follows: Clinical trials and observational studies comparing coronary dynamic roadmap to standard angiography during percutaneous coronary intervention, and all of studies focusing on the use of contrast medium. We placed no restrictions based on geographic location, language, age, gender, ethnicity, or publication date. Exclusion criteria were: (a) grey literature such as editorials, and other non-data literature, (b) meta-analyses and systematic reviews, (c) overlapping publications data, (d) if outcomes are not reported, (e) animal or in vitro studies.

A standardized data extraction form was utilized by tow reviewers to extract relevant data from the included studies. Three sets of data were extracted: the first set comprised the included studies’ characteristics, the second set included participants' demographic and baseline data, and the third set focused on our study outcomes. Categorical data were reported as frequencies and percentages, while continuous data were presented as means and standard deviations.

### 2.3 Study outcomes

Our objectives were to determine the differences between DCR-guided PCI and standard angiography PCI in terms of contrast medium volume, radiation time, dose-area product, air kerma, radiation dose, incidence of acute kidney injury (AKI), post-PCI estimated glomerular filtration rate (eGFR) and procedure success.

The dose-area product is a tool used to calculate the radiation dose in patients undergoing procedures or diagnostic tests that utilize x-ray radiation. This tool aids in assessing radiation risk and safety. Air Kerma is the amount of energy transferred from a photon to matter. Overall, air kerma is an essential parameter in clinical radiology, ensuring the safety and quality of radiological procedures.

### 2.4 Quality assessment

The risk of bias assessment for each included study was conducted independently by two reviewers. For randomized controlled trials, we used the Cochrane risk-of-bias tool, ROB2 [9]. Observational cohorts were assessed using the Newcastle-Ottawa Scale, NOS [10].

### 2.5 Data analysis

We used RevMen Web for our analysis. A random-effects model was applied, and the pooled analysis of the outcome means was conducted by calculating the standardized mean difference (SMD) with a 95 % confidence interval. A p-value of <0.05 indicates statistical significance. Acute kidney injury (AKI) was evaluated by calculating the incidence rate in both groups using the reported events along with the odds ratio with the 95 % confidence interval. Post-PCI eGFR was calculated using the mean difference.

Regarding heterogeneity, since the minimum number of studies required to provide a good sense is about 10, and our review only included 5 trials, we cannot have confidence that any of the statistics related to heterogeneity will be reliable [11]. A sensitivity analysis was conducted by one study removal method by RevMan to all of the included studies. We had intended to assess publication bias if the number of included trials approached or exceeded 10. Due to the limited number of studies (n = 5), formal publication bias assessment was not feasible. However, we aimed to minimize publication bias through a comprehensive search strategy in diverse sources to ensure the inclusion of all relevant studies.

## 3 Results

### 3.1 Study selection results

**Fig. 1:**
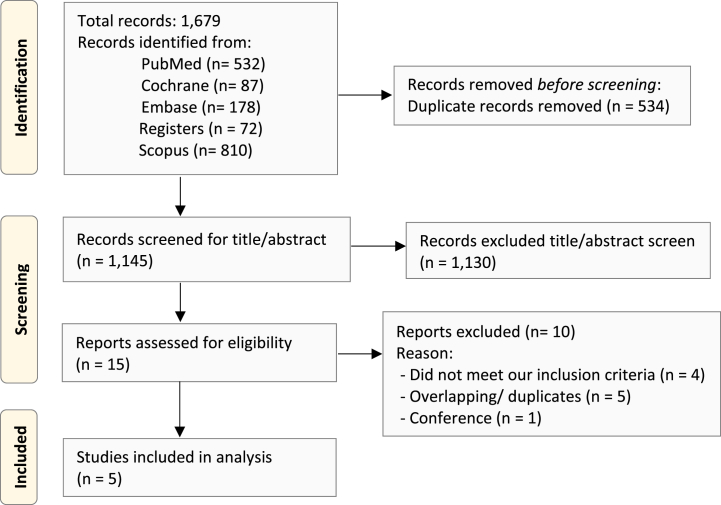
Study selection flow diagram.

Following the database search, we identified 1679 articles. Of these, only five were considered eligible for data extraction and analysis [[12], [13], [14], [15], [16]]. The selection process is illustrated in Fig. 1. Among the selected studies, three were cohort studies [[14], [15], [16]] and two were randomized controlled trials [12,13], all compared DCR with standard angiography PCI. The combined data included a total of 941 patients. A detailed overview of our included studies can be found in Table 1. The baseline characteristics of the study participants and procedural data can be found in Table 2.

**Table 1:** Characteristics summary of the included studies.

Study name	Piayda et al.,	Hennessey et al.,	Yabe et al.,	Bendary et al.,	Hirano et al.,
Year	2021	2024	2019	2023	2023
Region	Germany	Europe, Israel, and the USA	Japan	Egypt	Japan
Study type	RCT, open-label	RCT, unblinded	Cohort, retrospective	Cohort, prospective	Cohort, retrospective
Total No.	133	356	130	80	226
Outcomes	contrast medium usage, radiation time, dose-area product, air kerma, incidence of AKI, procedure success	contrast medium usage, radiation time, dose-area product, incidence of AKI, procedure success	contrast medium usage, radiation time, dose-area product, air kerma, procedure success	contrast medium usage, radiation time, dose-area product, air kerma, procedure success	contrast medium usage, radiation time, dose-area product, air kerma, incidence of AKI, procedure success

**Table 2:** Background characteristics of studies’ participants, n (%).

Study name	Piayda et al.,	Hennessey et al.,	Yabe et al.,	Bendary et al.,	Hirano et al.,
Sample size					
DCR	66	179	38	40	113
non-DCR	67	177	92	40	113
Age years, mean ± SD					
DCR		65.8 ± 10.8	74.84 ± 12.11	57 ± 10	75.54 ± 10.3
non-DCR		65.9 ± 10.9	73.52 ± 10.85	59 ± 10	72.39 ± 9.44
Gender, n (%)					
Male					
DCR		143 (79.9 %)	27 (71 %)	22 (55 %)	100 (88.5 %)
non-DCR		139 (78.5 %)	64 (69.5 %)	24 (60 %)	92 (81.4 %)
Female					
DCR		36 (20.1 %)	11 (29 %)	18 (45 %)	13 (11.5 %)
non-DCR		38 (21.5 %)	28 (30.5 %)	16 (40 %)	21 (18.6 %)
BMI, mean ± SD					
DCR		28.9 ± 5.7	23.01 ± 3.75	29 ± 4	24.13 ± 4.08
non-DCR		28.5 ± 4.8	23.73 ± 3.20	29 ± 4	24.46 ± 3.83
BSA, mean ± SD					
DCR	1.7 ± 0.6		1.63 ± 0.22		1.68 ± 0.18
non-DCR	1.6 ± 0.6		1.65 ± 0.21		1.68 ± 0.19
Hypertension					
DCR		135 (75.4 %)	26 (68.4 %)	27 (67.5 %)	100 (88.5 %)
non-DCR		124 (70.1 %)	73 (79.3 %)	27 (67.5 %)	91 (80.5 %)
Dyslipidemia					
DCR			27 (71 %)	37 (92.5 %)	91 (80.5 %)
non-DCR			68 (73.9 %)	36 (90 %)	88 (77.9 %)
Diabetes mellitus					
DCR		66 (36.9 %)	14 (36.8 %)	29 (72.5 %)	63 (55.8 %)
non-DCR		68 (38.4 %)	32 (34.7 %)	26 (65 %)	57 (50.4 %)
Chronic kidney disease					
DCR		18 (10.1 %)	8 (21 %)	10 (25 %)	113 (100 %)
non-DCR		26 (14.7 %)	30 (30.6 %)	0 (0 %)	113 (100 %)
Prior CABG					
DCR		12 (6.7 %)	0 (0 %)	1 (2.5 %)	7 (6.2 %)
non-DCR		15 (8.5 %)	0 (0 %)	4 (10 %)	6 (5.3 %)
Prior myocardial infarction					
DCR		55 (30.7 %)	3 (7.8 %)	10 (25 %)	53 (46.9 %)
non-DCR		54 (30.5 %)	11 (11.9 %)	16 (40 %)	44 (38.9 %)
eGFR, ml/min/1.73 m^2^,mean ± SD					
DCR		77.5 ± 19.7	64.45 ± 17.09		40.45 ± 9.09
non-DCR		75.5 ± 19.9	63.9 ± 18.28		42.95 ± 10.29
S.Cr. mg/dl, mean ± SD					
DCR			0.89 ± 0.31	0.98 ± 0.27	1.36 ± 0.28
non-DCR			0.89 ± 0.27	0.90 ± 0.14	1.32 ± 0.51

Abbreviations: BMI body mass index; BSA body surface area; eGFR estimated glomerular filtration rate. S.Cr. serum creatinine.

### 3.2 Quality assessment results

Due to the mixed design of the included studies, we used two types of bias assessment tools. Two of the studies were randomized controlled trials and were assessed using the Cochrane risk of bias tool version 2 (ROB2). Both Piayda et al. [12] and Hennessey et al. [13] were randomized controlled trials, and both had some concerns as they were open-label designs, and there was no mention of how randomization was carried out. The Newcastle-Ottawa Scale was used to evaluate the three cohort studies [[14], [15], [16]]. Studies scoring 7–9 were considered high quality, while studies scoring ≤6 were considered low quality. The results were as follows: two studies scored 8 [14,16], and one scored 7 [15].

### 3.3 Primary outcomes results

**Fig. 2:**
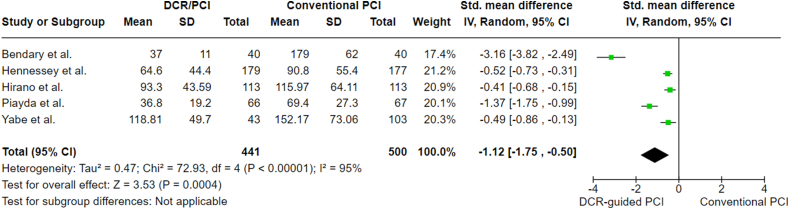
Contrast medium analysis.

The means of contrast medium volume was reported in all the included studies. There was a statistically significant decrease in SMD favoring DCR-guided PCI with an SMD of −1.12 (95 % CI: 1.75 to −0.50, p-value = 0.0004). This indicates that DCR-guided PCI was associated with lower usage of contrast medium. Looking at the graph, the study by Bendary et al. [15], which has the lowest mean compared to others, might suggest that heterogeneity is caused by it (I^2^ = 95 %). However, after conducting a sensitivity analysis and excluding the study by Bendary et al. [15], the effect remains significant with an SMD of −0.68 (95 % CI: 1.05 to −0.31, P = 0.0003), and heterogeneity remains high (I^2^ = 84 %). However, assessing heterogeneity is difficult due to the limited number of studies. Contrast medium analysis is presented in Fig. 2.

**Fig. 3:**
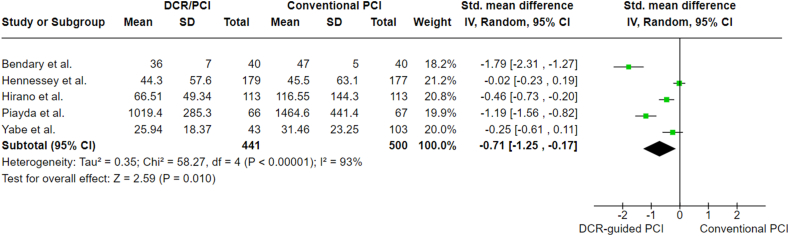
Dose-area product analysis'.

The dose-area product was reported in all the studies, and SMD was −0.71 (95 % CI: 1.25 to −0.17; p = 0.010), indicating lower dosage usage in DCR-guided PCI compared to standard angiography PCI. A sensitivity analysis, performed by removing one study tool, confirmed that the findings remained consistent. Heterogeneity remained high (I^2^ = 93 %) despite the sensitivity analysis. The analysis is presented in Fig. 3.

**Fig. 4:**
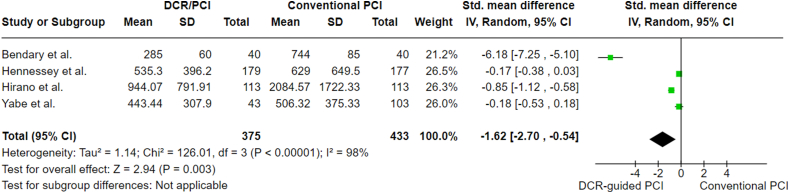
Air kerma analysis.

Air kerma was reported in four studies, with a standard mean difference (SMD) of −1.62 (95 % CI: 2.70 to −0.54; p = 0.003), indicating that DCR-guided PCI was associated with lower radiologic exposure compared to standard angiography PCI. A sensitivity analysis, conducted by removing the study by Bendary et al. [15], showed a mean difference of −0.40 (95 % CI: 0.85 to 0.05; p = 0.08), and heterogeneity remained high (I^2^ = 88 %). The analysis is reported in Fig. 4.

**Fig. 5:**
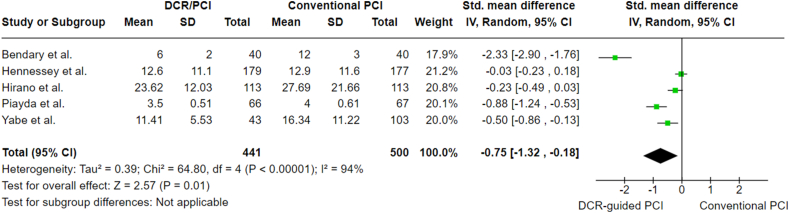
Radiation time analysis.

Radiation time was reported in all of the studies and it showed a statistically significant difference between DCR-guided PCI and standard angiography PCI with a SMD of −0.75 (CI 95 %: 1.32 to −0.18, p = 0.01). This indicates that DCR-guided PCI demonstrating a shorter duration in terms of radiation time. The results are illustrated in Fig. 5. Heterogeneity is high (I2 = 94 %) and remained the same despite the sensitivity analysis.

Acute kidney injury (AKI) was reported in three studies involving a total of 751 patients, with 358 in the DCR-guided PCI group and 357 in the standard angiography PCI group. The incidence of AKI in the DCR-guided PCI group was 1.11 %, compared to 3.36 % in the conventional PCI group. However, the odds ratio showed no statistically significant difference between the two groups (OR = 0.40; 95 % CI 0.13–1.27; p = 0.12). Post-PCI estimated glomerular filtration rate (eGFR) was mentioned in only two studies and did not show any statistical significance, with a mean difference of 4.66 (95 % CI: 8.43 to 17.76; P = 0.49). The results of AKI and eGFR analysis are presented in the Supplementary Figs. 1 and 2.

Finally, our analysis revealed no difference in procedural success rates between DCR-guided PCI and conventional PCI, with both achieving success rates exceeding 99 %.

## 4 Discussion

In our study, we focused on DCR-guided PCI and compared to standard angiography PCI in terms of their ability to reduce the use of contrast media during the procedure. Our analysis might have revealed the potential advantages of DCR-guided PCI over standard angiography PCI.

The amount of contrast medium used was lower in DCR-guided PCI, suggesting it might be a safer option for patients with high risks of CA-AKI. Furthermore, DCR-guided PCI showed some benefit in terms of lowering radiation exposure, with lower air kerma and dose-area product compared to standard angiography PCI. Additionally, DCR-guided PCI procedures were completed in less time, resulting in a lower overall radiation dose. The success rates for both procedures were over 99 %, may indicate that DCR-guided PCI can achieve similar procedural success rates to the conventional method.

There was no statistically significant difference in acute kidney injury between DCR-guided PCI and conventional PCI. However, DCR-guided PCI was associated with a lower contrast dose. Reducing the contrast volume to less than three times the estimated creatinine clearance, as is known, has been shown to prevent CA-AKI [17]. In patients with an estimated glomerular filtration rate (eGFR) of less than 30 ml/min/1.73 m2, a contrast-volume-to-creatinine-clearance (CV/CrCl) ratio more than 2 has been identified as an independent predictor of contrast-associated acute kidney injury (CA-AKI) [18].

DCR-guided PCI also have been used in complex anatomy. The study by Hennessey et al. observed that in patients with higher anatomical PCI complexity (SYNTAX score ≥4), the DCR arm utilized less contrast volume compared to the standard approach. However, for patients with lower complexity (SYNTAX score <4), there was no significant difference in contrast volume between the two methods [13].

In Hennessey et al., when evaluating the number of vessels involved in the procedure, whether single or multiple, the DCR-guided PCI group used less contrast media compared to the conventional PCI group. For single-vessel procedures, the DCR-guided PCI group used 58.2 ± 34.5 ml, while the conventional PCI group used 82.4 ± 52.7 ml. For multiple-vessel procedures, the DCR-guided PCI group used 110.2 ± 73.1 mL, compared to 130.4 ± 51.4 mL for the conventional PCI group [13].

### 4.1 Limitations and Implications for Future Research

Our review included only a small number of studies. The sample size of 941 patients, may still be insufficient to detect the benefits of it or to generalize the findings to all patient populations. The mix design of the included studies in our review is affected by the lack of randomized controlled trials in the literature; only two randomized clinical trial included, the rest are observational cohorts. This may introduce potential variability in the data due to differences in study protocols and patient selection criteria. Although patient selection bias might be present, particularly in the cohorts, where treatment decisions were not randomized and could be influenced by underlying patient conditions or physician preferences. There may be slight differences in the criteria used to assess CA-AKI by the included studies, and these should be interpreted cautiously.

## 5 Conclusions

Overall, this data suggests that DCR-guided PCI could be a potential alternative to conventional PCI, with the possibility of reduced contrast medium use, and lower radiation exposure, while maintaining comparable procedural success rates. Large randomized clinical trials that are powered enough to outline the advantages and differences between DCR-guided PCI and conventional methods in patients with complex anatomy and multivessel disease are needed. Furthermore, more studies are needed in patients with kidney disease, as this particular group would benefit the most from ultra-low contrast media imaging. Post-procedure serial measurements of daily eGFR and serum creatinine levels would provide valuable information.

## CRediT authorship contribution statement

**Mohammad Al Hayek:** Writing – original draft, Visualization, Methodology, Formal analysis, Data curation, Conceptualization. **Ibrahem A. Beshr:** Writing – review & editing, Writing – original draft, Supervision, Software, Methodology, Data curation, Conceptualization. **Mohammed S. Beshr:** Writing – review & editing, Writing – original draft, Supervision, Methodology, Formal analysis, Data curation, Conceptualization.

## Data availability

Data is available upon reasonable request from the authors.

## Funding

None.

## Declaration of competing interest

The authors have nothing to declare.

## List of abbreviations

PCI: Percutaneous coronary intervention
CA-AKI: Contrast associated acute kidney injury
DCR: Dynamic Coronary Roadmap
AKI: Acute kidney injury (AKI)
IVUS: Intravenous ultrasound
SMD: Standardized Mean Difference
eGFR: estimated glomerular filtration rate
