# Supplementary material for: Dynamic coronary roadmap-guided PCI reduces contrast volume and radiation time compared to standard angiography PCI: A meta-analysis
Source: Heliyon. 2024 Dec 30;11(1):e41557. doi: 10.1016/j.heliyon.2024.e41557 (PMC11750551; doi:10.1016/j.heliyon.2024.e41557)

Supplmentary materials

This supplementary metarials for the following paper:

Dynamic Coronary Roadmap (DCR-guided) compared standard angiography PCI: a systematic review and meta-analysis

Tables 1. Databse search terms stratgey 02

Table 2. Risk of bias assessment results 03

Figure 1. Acute kidney injury (AKI) 03

Figure 2. Post-PCI estimatred glomurlar filtration rate (eGFR) 03

Tabels 1. Serach terms strategy

**Search Query PubMed**		**N**
**#1**	dynamic coronary roadmap OR DCR OR dynamic roadmap OR roadmap OR coronary roadmap	16,575
**#2**	"Percutaneous Coronary Intervention"[Mesh] OR "PCI"[tw] OR "percutaneous coronary intervention*"[tw] OR "coronary Intervention*"[tw] OR "percutaneous coronary revascularization*"[tw] OR "Coronary Angiography"[Mesh] OR "coronary angiograph*"[tw]	161,588
**#3**	"Coronary Artery Disease"[Mesh] OR "coronary artery disease"[tw] OR CAD[tw] OR "coronary arteriosclero*"[tw] OR "coronary atherosclero*"[tw]	174,614
**#4**	"Contrast medium" OR "contrast reduction" OR "Contrast Media"[Mesh] OR contrast[tw] OR "contrast agent"[tw]	122,0455
**#5**	#1 AND (#2 OR #3 OR #4)	538

**Search Query Embase (title, abstract, author key)**		**N**
**#1**	dynamic coronary roadmap OR DCR OR dynamic roadmap OR roadmap OR coronary roadmap	10,800
**#2**	pci OR 'percutaneous coronary intervention*' OR 'coronary intervention*' OR 'percutaneous coronary revascularization*' OR 'coronary angiograph*'	221,279
**#3**	"'coronary artery disease' OR cad OR 'coronary arteriosclero*' OR 'coronary atherosclero*'	41,0941
**#4**	'contrast medium':ti,ab,kw OR 'contrast reduction':ti,ab,kw OR 'contrast media':ti,ab,kw OR 'contrast agent':ti,ab,kw	66,339
**#5**	#1 OR #2 OR #3	62,0497
**#6**	#4 AND #5	178

**Search Query Cochrane**		**N**
**#1**	dynamic coronary roadmap OR DCR OR dynamic roadmap OR roadmap OR coronary roadmap	3,249
**#2**	PCI OR Percutaneous Coronary Intervention OR PCI OR percutaneous coronary intervention OR coronary Intervention OR "percutaneous coronary revascularization OR Coronary Angiography OR coronary angiography	29,635
**#3**	Coronary Artery Disease OR coronary artery disease OR CAD OR coronary arteriosclerosis OR "coronary atherosclerosis	32,182
**#4**	Contrast medium OR contrast reduction OR Contrast Media OR contrast OR contrast agent	51,492
**#5**	#1 AND (#2 OR #3 OR #4)	105

**Search Query clinicaltrials.gov**		**N**
**#1**	dynamic coronary roadmap OR DCR OR dynamic roadmap OR roadmap OR coronary roadmap	72

Table 2. Risk of Bias Assessment of Included Studies

**Cochrane risk-of-bias tool, ROB2**						
**Study**	**D1**	**D2**	**D3**	**D4**	**D5**	**Overall**
Piayda et al, 2021	Some concerns	low	low	low	low	Some concerns
Hennessey et al, 2024	Some concerns	low	low	low	low	Some concerns
**Newcastle-Ottawa Scale, NOS**						
**Study**	**Selection**	**Comparability**	**Exposure**	**Total**		
Yabe et al, 2019	4	2	2	8		
Bendary et al, 2023	3	2	2	7		
Hirano et al, 2023	4	2	2	8		

Figure 1. Acute kidney injury (AKI) analysis () and post-PCI estimatred glomurlar filtration rate (eGFR)

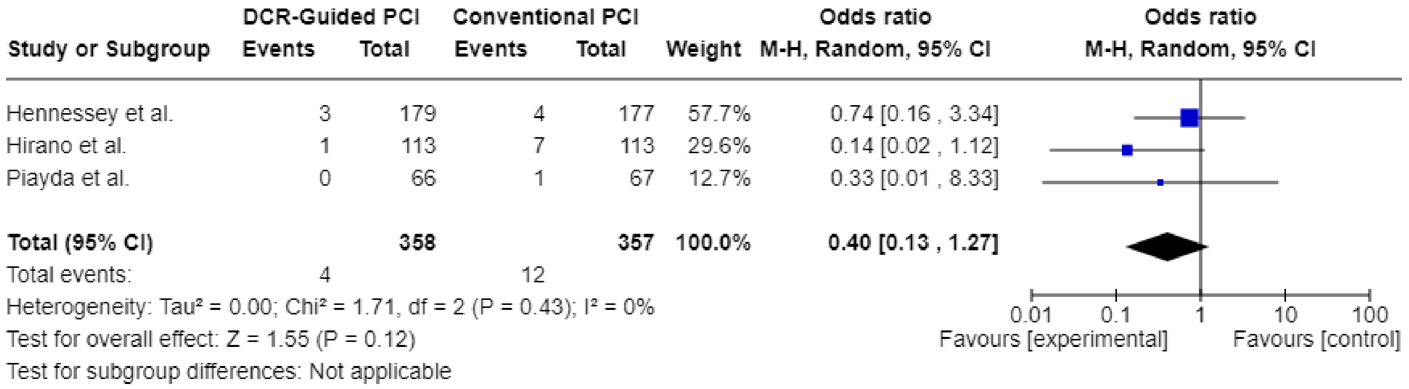

Figure 2. Post-PCI estimatred glomurlar filtration rate (eGFR)

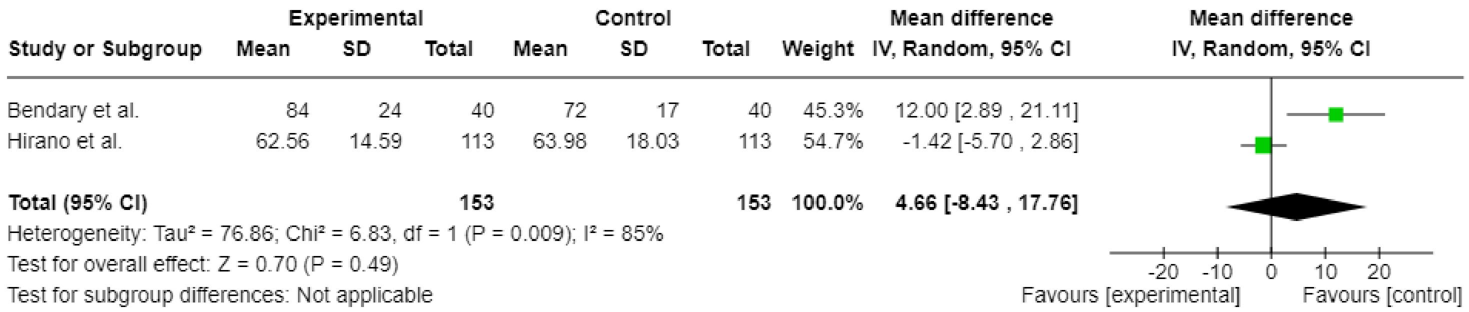

Supplement: Multimedia component 1 [file mmc1.pdf]
